# Feasibility of an Intervention Targeting Health through Exergaming as an Alternative to Routine Treatment (FIT HEART): protocol for a non-randomised two-armed pilot study

**DOI:** 10.1186/s40814-022-01068-2

**Published:** 2022-06-11

**Authors:** Sharlene Kaye, Amy Lewandowski, Mitchell Dunne, Julia Bowman, Vicki Archer

**Affiliations:** 1 Research Unit, Justice Health and Forensic Mental Health Network, Long Bay Complex, Roundhouse, 1300 Anzac Parade, Malabar, NSW 2036 Australia; 2 National Drug and Alcohol Research Centre, UNSW Sydney, Sydney, Australia

**Keywords:** Exergaming, Active videogaming, Physical activity intervention, Mental health, Inpatient, Cardiovascular, Cardiometabolic risk

## Abstract

**Background:**

Despite elevated risk of cardiometabolic disease among those with serious mental illness, and widespread recognition that physical activity interventions are required, there are multiple barriers to implementing typically recommended physical activity programmes in secure inpatient settings. Due to low mood, negative symptoms and poor socio-occupational functioning, psychiatric inpatients often lack motivation to engage in physical activity programmes. Moreover, regular access to outdoor spaces and exercise equipment is limited. As such, there is a need for novel physical activity interventions that are suitable for secure settings. This study aims to investigate the feasibility, acceptability and potential effectiveness of an intervention (exergaming) to promote physical activity among patients in a secure mental health setting.

**Methods:**

This non-randomised, two-arm pilot study will employ a pre-test/post-test parallel group design, comparing the exergaming intervention with a “routine treatment” control. Two high-secure, sub-acute wards in the Long Bay Hospital Mental Health Unit will be non-randomly allocated to either the exergaming intervention or the “routine treatment” control group.

The intervention group will receive a 12-week programme comprising three 30-min exergaming sessions per week using various Xbox Kinect^TM^ activity-based games designed to simulate moderate intensity exercise. The “routine treatment” group will continue to receive the standard model of care delivered by the Justice Health and Forensic Mental Health Network. Accelerometers will be distributed to all participants to collect daily energy expenditure, number of steps taken, intensity of physical activity and heart rate data throughout the study.

The primary outcomes are (1) intervention feasibility and acceptability, and (2) baseline to post-intervention changes in physical health outcomes (levels of physical activity; cardiovascular fitness; clinical measures of cardiometabolic risk). Secondary outcomes are baseline to post-intervention changes in mental health outcomes (depression, anxiety, stress, positive psychiatric symptoms). Outcomes will be assessed at baseline, mid-intervention, and post-intervention.

**Discussion:**

This research will contribute to evidence-based practice in the care of patients with serious mental illness: a vulnerable population with complex physical and mental health needs and a markedly elevated risk of cardiovascular disease. The findings will inform cardiovascular health promotion strategies and the implementation of physical activity interventions in secure inpatient settings.

**Trial registration:**

ANZCTR, ACTRN12619000202167. Registered on 12 February 2019, https://www.anzctr.org.au. ANZCTR mandatory data items comply with the minimum dataset requirements of the World Health Organisation (WHO). The ANZCTR contributes trial registration data to the WHO International Clinical Trials Registry Platform (WHO ICTRP).

**Supplementary Information:**

The online version contains supplementary material available at 10.1186/s40814-022-01068-2.

## Background

The adverse metabolic effects associated with psychotropic medications used to treat mental illness are well-documented. Clinically significant weight gain is commonly observed following the commencement of pharmacotherapy for mood and psychotic disorders—particularly treatment with antipsychotics. Disturbances in glucose and lipid metabolism, either secondary to weight gain or via direct mechanisms, are also associated with such medications [1, 2]. In addition to lifestyle factors (e.g. smoking, poor diet, lack of physical activity), these medication side effects increase the risk of metabolic syndrome (MetS) and, in turn, type 2 diabetes and cardiovascular disease. Accordingly, the prevalence of MetS is elevated among those treated with psychotropic medication compared to the general population [3].

There is a growing evidence base for the role of physical activity in improving health outcomes in psychiatric populations, with systematic reviews and meta-analyses of the literature suggesting that exercise interventions can effectively improve cardiorespiratory fitness and reduce cardiometabolic risk [4–6]. Implementing lifestyle interventions in a psychiatric inpatient setting, however, particularly that of a secure mental health unit, presents many challenges. Environmental barriers to physical activity include restricted time in outdoor and communal spaces and limited access to exercise equipment. Moreover, psychiatric inpatients often lack the motivation to engage in physical activity secondary to low mood, negative symptoms and poor socio-occupational functioning. As such, there is a need for novel physical activity interventions suitable for patients in secure settings that will overcome such barriers.

Research into the utility of “exergaming” as a form of physical activity has shown promising results. Exergaming refers to active video games that use the player’s movement to control the game. Laboratory-based experimental studies of the acute effects of exergaming have demonstrated increased energy expenditure, heart rate and oxygen consumption at an exertion level equivalent to “moderate intensity” exercise [7, 8], depending on the nature of the game and extent of lower body involvement [9]. Intervention studies indicate that exergaming results in increased energy expenditure, improved cardiovascular fitness and increased muscle strength, in addition to a decrease in body weight, waist circumference, and body fat percentage [10–12]. Positive effects on mood, motivation to exercise and cognitive functioning have also been demonstrated [2, 13]. While research among psychiatric populations is limited, pilot data on feasibility, acceptability, adherence and safety validate the integration of exergaming into physical activity programmes for people with serious mental illness. Specifically, intervention trials among people with schizophrenia have demonstrated active videogaming to be an acceptable and enjoyable alternative to other forms of physical activity, with high adherence and retention rates observed among participants [13–18].

Whilst previous research suggests that exergaming-based interventions are feasible and acceptable among psychiatric patients in an outpatient setting [14, 18, 19], there has been no research investigating the feasibility and acceptability of exergaming in an inpatient setting. Moreover, the clinical effectiveness of such interventions in promoting the cardiovascular health of this particular patient population has not yet been investigated. The few studies investigating the health benefits of such interventions for psychiatric populations have typically used a predominantly outpatient sample and have only focused on the amount and frequency of physical activity and functional fitness (e.g. balance, strength, flexibility, mobility) as study outcomes, rather than cardiorespiratory fitness measures and cardiometabolic parameters [14, 18, 19].

### Study aims and objectives

This pilot study aims to investigate the feasibility, acceptability and potential effectiveness of a novel intervention (exergaming) to promote physical activity among patients of a secure mental health unit. The results will inform the design and methodology of a subsequent randomised controlled effectiveness trial.

The specific objectives of the study are to:

1. Determine whether the pilot exergaming programme (FIT HEART) is a feasible intervention for improving physical activity levels among patients in a secure mental health unit, as measured by recruitment rates, retention in the programme, protocol adherence, and patient and staff acceptability ratings.
2. Assess whether participants who receive the FIT HEART programme demonstrate greater improvements in physical activity levels and clinical physical health measures (anthropometry; blood parameters; blood pressure), compared to those who receive routine treatment only.

Based on the emerging evidence, it is hypothesised that the FIT HEART programme exergaming will be a feasible and acceptable form of physical activity intervention among this patient group. Findings on the potential effectiveness of the intervention as an adjunct to routine care in providing physical and mental health benefits to patients will inform hypotheses for a future randomised trial.

## Methods

### Study design

The proposed research will be conducted as a non-randomised, two-arm pilot study comparing an exergaming intervention with a “routine treatment” control. The study will employ a pre-test/post-test parallel group design. Two sub-acute wards from the Long Bay Hospital Mental Health Unit will be non-randomly allocated to either the intervention or control group. Allocation will be based on available facilities (i.e. space, audiovisual equipment) to implement the intervention.

The study duration will be 24 months and will be reported in accordance with guidelines for reporting the protocols of pilot and feasibility studies [20], adapted for a non-randomised design [21], using relevant items from the Standard Protocol Items: Recommendations for Interventional Trials (SPIRIT) checklist [22] (see Additional file 1).

### Setting

The study will be conducted in two sub-acute units of the Long Bay Hospital Mental Health Unit, located at the Long Bay Correctional Complex in New South Wales (NSW), Australia. The Long Bay Hospital Mental Health Unit is a 40-bed inpatient facility that provides specialty mental health services for correctional and/or forensic patients within the Justice Health and Forensic Mental Health Network (JHFMHN). The two sub-acute units house 15 male patients each.

### Participants

The sample will comprise 60 adult patients admitted to the Long Bay Hospital Mental Health Unit (intervention group: 30 patients; control group: 30 patients). All patients aged 18 years and over that are currently admitted to the Long Bay Hospital Mental Health Unit will be given the opportunity to participate in the study, subject to meeting eligibility criteria.

#### Eligibility criteria

Potential participants will be screened for eligibility by members of the FIT HEART project team. The team is based at the JHFMHN Research Unit and comprises experienced researchers with qualifications in nursing, psychology, forensic mental health, and exercise physiology. Screening will entail the following: (i) assessment by the FIT HEART project team of capacity to provide informed consent; (ii) assessment by the treating team of security risk and risk factors that could prevent participants being issued an accelerometer; (iii) screening by the FIT HEART project team and clinical assessment by a medical officer of physical contraindications to exercise.

Patients will be excluded from the study if they meet the any of the following exclusion criteria:

1. Risk factors that could prevent patients being issued an accelerometer (e.g. at risk of self-harm; acute paranoid delusions).
2. Inability to provide informed consent due to acute mental illness, severe cognitive impairment or severe intellectual disability.
3. Insufficient English fluency to provide informed consent and/or complete the pre- and post-test interviews.
4. Unable to participate due to high security risk, as determined by the treating team.
5. Physical contraindications to exercise.

### Eligibility screening and consent procedures

The study will be introduced to patients and staff from the selected units via an oral presentation delivered by the FIT HEART project staff and posters containing information on the nature and purpose of the study which will be displayed in the units.

A pre-screening risk assessment for patients in both the intervention and control units will be conducted by a member of the treating team to ascertain whether the patient is a high security risk (exclusion criterion 4) or has any risk factors that could prevent them being issued an accelerometer to wear during the study period (exclusion criterion 1). Any patients deemed to have risk factors that preclude them from wearing an accelerometer or that are identified as a high security risk will not undergo further screening and will be excluded from the study.

All potential participants eligible for further screening will be provided with a participant information sheet (PIS) detailing the nature and aims of the study at an appropriate level of readability, determined by the Flesch Reading Ease formula [23]. Those who are interested in being screened for study eligibility will be assessed for their capacity to consent (exclusion criterion 2) to the screening process, using the University of California, San Diego Brief Assessment of Capacity to Consent (UBACC) [24] tool. If deemed capable, participants will be asked to provide written consent for all screening procedures.

**Fig. 1:**
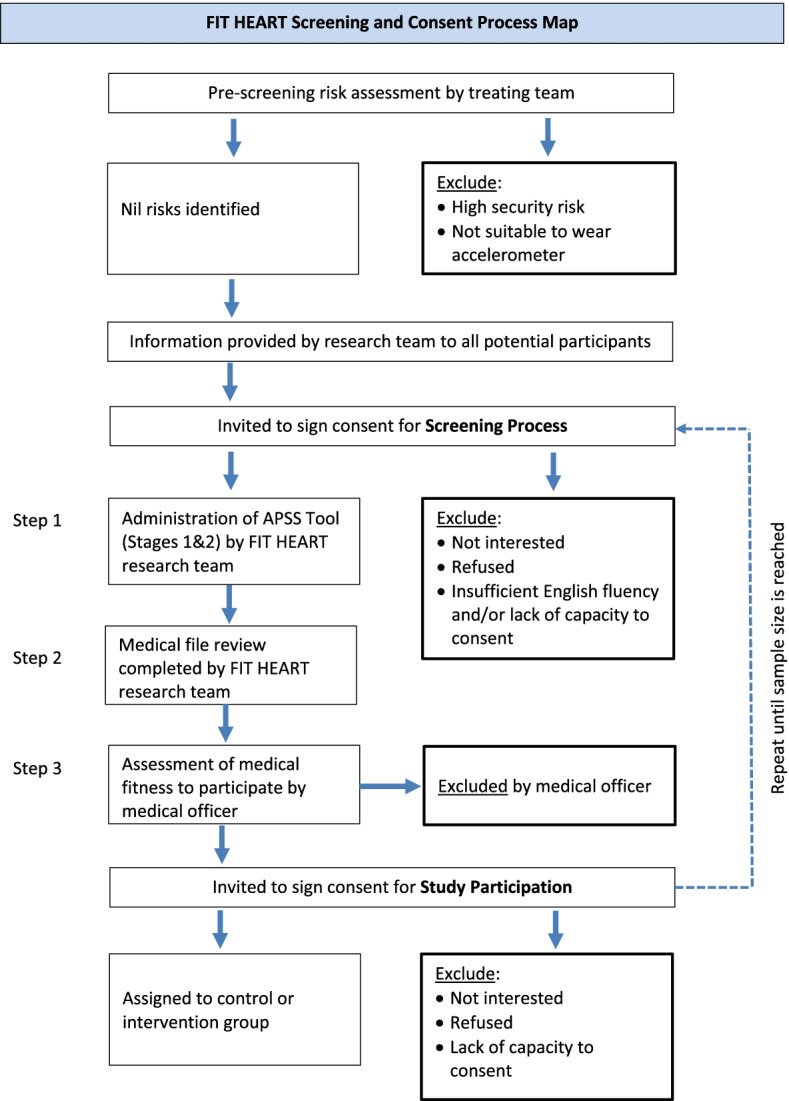
Flowchart of participant eligibility screening and consent

The screening procedure will comprise the following steps (Fig. 1):

1. An assessment of medical conditions that may increase the risk of an adverse event. This will involve completion of a modified version of the Adult Pre-Exercise Screening System (APSS) Tool, a three-stage screening instrument developed by Exercise and Sport Science Australia, Fitness Australia and Sports Medicine Australia [25]. This questionnaire identifies symptoms and/or diagnoses of cardiovascular, metabolic, respiratory and other diseases, as well as musculoskeletal conditions that may be exacerbated by physical activity (stage 1). The tool also considers risk factors (e.g. age, family history of cardiovascular disease, current activity levels and smoking) before categorising the participant as either low or moderate risk (stage 2). The APSS Tool will be administered by one of the research team.
2. A clinically trained member of the research team will conduct a review of the patient’s medical file identifying current medical and psychiatric conditions, current and past medications and electrocardiogram results.
3. A medical officer will conduct an assessment of medical fitness to participate in an exercise programme (exclusion criterion 5), based on risk factors identified during administration of the APSS Tool, contraindications to exercise arising from prescribed medications, and known medical/psychiatric history. If the medical officer deems the participant inappropriate, they will be excluded from the study.

Patients who screen eligible for participation will be asked to provide further written consent for all study procedures, with their capacity to consent assessed a second time with the UBACC tool. One of the research team will witness the signing of all consent forms. Reasons for participant ineligibility and refusal to participate for eligible patients will be documented in a screening log.

### Interventions

#### Exergaming

Participants enrolled in the intervention group will receive a 12-week exergaming programme, delivered by members of the FIT HEART research team who will undergo project-specific training and will be provided with a manual detailing all study procedures. The programme comprises three 30-min exergaming sessions per week for 12 weeks using activity-based games designed to simulate moderate intensity exercise. The exergaming programme will be delivered via Xbox Kinect^TM^ video gaming consoles, with face-to-face supervision of all sessions provided by the FIT HEART research team, nursing staff and unit-based Corrective Services NSW officers. The Xbox Kinect^TM^ system has a large variety of games available, all of which can be played without any equipment (e.g. hand-held controllers). The absence of controllers will minimise the risk of harm to the participants and others. All games to be used in the sessions have been tested by the research team to ensure that a moderate intensity heart rate can be achieved. Selected games include those based on sports, fitness and adventure games (e.g. Kinect Sports, Kinect Adventures, MotionSports Adrenaline, Your Shape: Fitness Evolved, Kinect Rush), dance/rhythm simulation (e.g. Black Eyed Peas Experience, Dance Central) and “party” games (e.g. Raving Rabbids: Alive and Kicking). Each 30-min session will be preceded by a 10-min warm-up utilising one of the lower intensity exergames (e.g. ten-pin bowling, Kinect Joy Ride) and will be followed by a 5-min cool-down, with a choice of stretches designed by an Exercise Physiologist. At 10-min intervals during each exergaming session, participants will be asked to rate their perceived intensity of physical activity using the OMNI-Walk/Run Scale of Perceived Exertion [26] and report the current heart rate displayed on their accelerometer. These measurements will be recorded by the supervising member of the research team. If a participant’s heart rate is not in the moderate intensity range, or if they are seen to be not participating in the game, they will be encouraged to try and increase their intensity or swap to another game.

The intervention protocol is based on current Australian guidelines for physical and sedentary activity. The Australian Government Department of Health recommend that adults (18–64 years) accumulate 150–300 min of moderate intensity or 75–150 min of vigorous intensity physical activity (or an equivalent combination of moderate and vigorous activity) per week. For those who are not currently engaged in regular physical activity, however, a gradual increase in frequency and intensity is recommended [27]. The proposed protocol is also supported by international clinical guidelines for the treatment of mental illness which recommend physical activity programmes consisting of thrice-weekly exercise sessions for a duration of 12 weeks as a low-intensity psychosocial intervention for subthreshold or mild-moderate depression [28].

#### Routine treatment

Enrolled participants on the control unit will receive “routine treatment”, defined as the standard model of care provided by JHFMHN (i.e. usual care). Structured physical activity (e.g. organised walking, gym sessions) over the 14 weeks of monitoring that occurs outside of the assessment periods will be recorded.

### Outcomes

#### Primary outcomes

1. Intervention feasibility and acceptability
2. Baseline to post-intervention changes in physical health outcomes:
  - Levels of physical activity
  - Cardiovascular fitness
  - Anthropometric measures of cardiometabolic risk: weight, body mass index (BMI), waist and hip circumference
  - Biomarkers of cardiometabolic risk: blood pressure, blood glucose levels, lipid profiles

#### Secondary outcomes

1. Baseline to post-intervention changes in mental health outcomes:
  - Symptoms of depression, anxiety and stress
  - Positive psychiatric symptoms

### Participant timeline

The study will be conducted over 14 weeks, including a 12-week intervention period. Participants will be assessed on three occasions: baseline, mid-intervention (week 6) and post-intervention (weeks 13–14). In cases where a participant is discharged from the control or intervention unit, or discontinues their participation, prior to completion of the intervention period (i.e. before week 12), post-intervention assessments will be administered, where feasible. Full details of the timeline and schedule of assessments are presented in Table 1.

**Table 1:** Schedule of enrolment, intervention and assessments

	Study period	***Pre-screening***	***Screening***	***Baseline***	***Intervention*** (exergaming or usual care)	***Intervention mid-point***	***Early discontinuation***	***Post-intervention***
	Timepoint	Day -14	Day -14 to day -7	Week 0	Weeks 1–12	Week 6	Discontinuation prior to week 12	Weeks 13–14
**Assessment/procedure**	Advertising of study	x						
	Pre-screening risk assessment	x						
	Inclusion and exclusion criteria		x					
	Participant information sheet		x					
	Informed consent form		x					
	Adult Pre-Exercise Screening System (APSS) (stages 1 and 2)		x					
	Assessment by medical officer of medical fitness to participate in physical activity		x					
	Baseline interview (demographics; incarceration history; perceived health status; psychiatric history)			x				
	Clinical assessment of cardiometabolic risk (APSS stage 3) • Body mass index (BMI) • Waist and hip circumference • Resting blood pressure • Lipid profile (total cholesterol, HDL, LDL, triglycerides) • Liver function • Blood glucose (HbA1c; fasting glucose)			x			x	x
	Current Community Assessment of Psychic Experiences-Positive Scale (Current CAPE-P15)			x				x
	Depression Anxiety Stress Scale (DASS-21)			x		x	x	x
	Simple Physical Activity Questionnaire (SIMPAQ)			x		x	x	x
	OMNI-Walk/Run Scale of Perceived Exertion*				x			
	YMCA Step Test (VO_2_max testing)			x			x	x
	Dynamic Appraisal of Situational Aggression (DASA)				x			
	Adverse event check				x		x	x
	Compliance and adherence assessments				x			
	Participant acceptability/perceived utility survey*						x	x
	Staff acceptability/feasibility survey*						x	x

* Intervention group only

### Sample size

As this is a pilot study, power-based sample size calculations have not been conducted. It is anticipated that findings from this study will inform sample size and power calculations for a subsequent definitive randomised controlled trial (RCT). The proposed sample size of 30 participants per study arm, a total of 60 participants, is based on recommended sample sizes for pilot studies preceding a main trial powered to detect a small to medium effect size (i.e. 0.1–0.3) [29] and is consistent with the median intended sample size of 30 participants per arm in registered pilot and feasibility trials [30]. Moreover, this sample size is sufficient to estimate recruitment and attrition rates ahead of a larger RCT across multiple sites. While there are no previous studies using a similar 12-week protocol among a wholly inpatient population to guide estimates of attrition, based on the attrition rates of studies among outpatient and non-psychiatric samples (7–19%) [11, 17, 18], we have conservatively allowed for a 20% attrition rate when considering our intended sample size.

### Recruitment strategy

As the number of potential participants at each unit will be limited to the number of beds in the ward, a rolling recruitment strategy (i.e. ongoing recruitment over the study period) will be employed. This strategy will create ongoing recruitment opportunities due to patient flow through the units and help minimise the effects of attrition on the planned sample size. Recruitment and retention will be monitored at regular intervals (i.e. weekly), with recruitment projections revised accordingly.

### Data collection

Baseline and post-intervention interviews will assess all primary and secondary outcomes, with the exception of intervention feasibility and acceptability which will be assessed on completion or discontinuation of the intervention, and are expected to take 60 min to complete. Mid-intervention interviews will measure self-reported levels of physical activity and symptoms of depression, anxiety and stress over the preceding week and are expected to take 20 min to complete. All interviews will be conducted by trained research staff, with the clinical assessments conducted by a research nurse.

#### Baseline assessment

Consenting eligible participants in both the intervention and control groups will complete a structured baseline interview administered by one of the research team. The baseline interview will collect information on demographic characteristics (age, gender, ethnicity, level of education, employment prior to incarceration, marital status), incarceration history (as a juvenile and adult), perceived general health status, psychiatric history as indicated by diagnosed mental health disorders (including substance use disorders), and previous exergaming experience (intervention group only). It will also will include the administration of validated instruments to assess potential effectiveness outcomes related to participants’ physical and mental health and a clinical assessment of cardiovascular and metabolic risk.

#### Primary outcome measures

##### Study feasibility

Feasibility will be measured by the number of participants recruited to the study; participation refusal rates; participant compliance and adherence (i.e. number, length and frequency of intervention sessions attended; compliance with wearing an accelerometer); rates of retention/attrition; occurrence of adverse events (e.g. injury, event related to psychotropic medication, abnormal increase in heart rate during intervention); fidelity of intervention delivery.

Recruitment data will be recorded in screening and enrolment logs, consent forms and study records. Data on intervention compliance, adherence, safety and fidelity of delivery will be recorded on a weekly basis.

In cases where enrolled participants discontinue before completion of the intervention period, reasons for early withdrawal will be documented. Participant discontinuation of the programme due to discharge from the unit will also be documented.

##### Acceptability of intervention

**Acceptability of intervention to participant:** Participant acceptability of the exergaming intervention will be assessed via post-intervention evaluation surveys (approximately 5 min) administered by research staff. Acceptability questions have been adapted from Kimhy et al. [17] and include questions on self-reported satisfaction with the intervention, ease of use of the exergaming system, and motivation to use exergaming as a form of physical activity in the future. Perceived utility of the intervention will be measured by asking participants about the impact of exergaming on their physical and mental health.

**Acceptability of intervention to staff at the study sites:** Acceptability of the exergaming intervention from the service perspective will be assessed via a post-intervention evaluation survey (approximately 5 min) using questions based on the theoretical framework of acceptability proposed by Sekhon et al. [31]. Areas assessed include staff perceptions of the intervention content and delivery, benefit for patients, and barriers to implementation and uptake.

##### Potential effectiveness of intervention

The potential effectiveness of the exergaming intervention will be assessed by measuring baseline to post-intervention changes in physical health outcomes:

i. Levels of physical activity
ii. Cardiorespiratory fitness
iii. Measures of cardiometabolic risk

**Physical activity: **Subjective levels of physical activity in the preceding week will be measured at baseline, mid-intervention and post-intervention using the Simple Physical Activity Questionnaire (SIMPAQ), a 5-item tool designed to assess physical activity and sedentary behaviour among people with mental illness [32]. The SIMPAQ measures self-reported activity over the previous week, including time spent in bed, sedentary time, structured exercise participation, and incidental or non-structured physical activity. In a validation study across 23 countries, the SIMPAQ was administered in a diverse sample of psychiatric patients, in both inpatient and outpatient settings, and found to be a reliable and valid tool for the assessment of self-reported physical activity [32].

Objective measures of daily physical activity during the study period will be obtained via accelerometry data. Following the baseline interview, all participants in both the intervention and comparison groups will be issued with an wrist-worn activity tracker (Fitbit® Charge 3) which they will be asked to wear the for the duration of the intervention period and up until the post-intervention assessments, totalling 14 weeks of activity monitoring. The Fitbits will only be worn whilst participants are out of their cell/room and participants will be required to check them in and out each day. Accelerometry data, obtained at 1-min intervals, will include number of steps taken, heart rate, energy (i.e. kilojoule) expenditure, and physical activity intensity as indicated by metabolic equivalent of task (METs) thresholds [33]. All participants will be provided with a weekly summary of their progress which will include their average steps over the preceding week, average heart rate, activity intensity levels (i.e. low, average, high) and average energy expenditure.

**Cardiorespiratory fitness:** Cardiorespiratory fitness at baseline and post-intervention will be determined via submaximal exercise testing using a modified version of the YMCA 3-min Step Test [34]. The modified YMCA step test protocol, which measures post-exercise heart rate recovery, has been shown to be a valid method of estimating VO_2_max [34–36], a measure of maximal oxygen consumption during exercise, with higher values of VO_2_max indicating higher levels of cardiorespiratory fitness.

**Cardiometabolic risk:** A clinical assessment of cardiovascular and metabolic risk factors, using the APSS (stage 3), will be conducted at baseline and post-intervention. Stage 3 of the APSS Tool obtains measurements of cardiovascular and metabolic risk factors (i.e. anthropometry, resting blood pressure, blood measures). This will entail measuring participants’ weight, BMI, waist circumference, hip circumference, and resting blood pressure. Blood samples will be obtained for testing of lipid profiles (total cholesterol, high-density lipoprotein (HDL), low-density lipoprotein (LDL), triglycerides), blood glucose levels (HBA1c, fasting glucose), and liver function. Capillary blood samples for lipid and glucose testing will be collected via fingerprick and analysed through a point-of-care testing machine. Blood for liver function testing will be collected via venous sampling.

#### Secondary outcome measures

##### Potential impact of intervention on mental health

The potential impact of the exergaming intervention will be assessed by measuring baseline to post-intervention changes in mental health outcomes:

**Depression, anxiety and stress:** Symptoms of depression, anxiety and stress will be measured using the Depression Anxiety Stress Scale (DASS-21). The DASS-21 is a 21-item self-report measure of symptoms of depression, anxiety and stress over the previous week [37]. Higher scores on the DASS-21 indicate a greater severity of symptoms. Assessments will be conducted at baseline, intervention mid-point and post-intervention.

**Positive psychiatric symptoms:** Positive psychiatric symptoms will be assessed at baseline and post-intervention using the Current Community Assessment of Psychic Experiences-Positive Scale (CAPE-P15) [38], a 15-item self-report scale that measures the frequency of psychotic-like experiences over the preceding 3 months. The CAPE-P15 comprises three sub-scales (persecutory ideation, bizarre experiences, perceptual abnormalities), with higher scores indicating more frequent symptomatology.

#### Other measures

##### Exercise intensity

Perceived exertion during exergaming sessions will be measured using the OMNI-Walk/Run Scale of Perceived Exertion assessment for adults [26]. Using the OMNI walking/running pictorial as a visual aid, participants will be asked to rate how hard or easy they perceive the exergaming session to be, based on their perceived levels of exertion. The OMNI-Walk/Run Scale includes numerical categories from 0 to 10, representing a range of exertion levels from “extremely easy” (0) to “extremely hard” (10). OMNI ratings of perceived exertion (RPE) have been validated against objective physiological measures of exercise intensity [26].

In order to ensure that participants do not exceed 85% of their maximum heart rate, as determined using an age-based formula (220−age of participant × 0.85), heart rate monitoring will be conducted at 1-min intervals during the baseline and post-intervention step tests and 10-min intervals during each exergaming session. If, at any time during the step tests, a participant’s heart rate approaches 85% of their maximum heart rate, the test will be discontinued. If this occurs during an exergaming session, a lower intensity game will be used for the remainder of the session.

##### General health status

Participants’ perception of their physical health status will be measured during the baseline and post-intervention interviews by asking them to rate their general health as “excellent”, “very good”, “good”, “fair” or “poor”. Self-reported physical health status has been found to be a useful indicator of chronic disease risk and prevalence among the prison population [39].

##### Study participation experience

All participants will be asked about their experience of participation in the study. Specifically, they will be asked whether they enjoyed being part of this study; whether they found wearing the Fitbit comfortable/uncomfortable; if they spent less time sitting while wearing the Fitbit; whether their usual daily routine had changed since being part of the study; if the weekly progress Fitbit information they were provided was useful; and, what things they liked most and least about the study. They will also be given the opportunity to provide comments or suggestions about the project.

### Data management

Data collected at each assessment occasion will be de-identified, with a unique alphanumeric study identifier (ID) created for each participant. All data will be entered into an IBM SPSS database (version 26) for storage and statistical analysis.

De-identified accelerometry (Fitbit) data will be transferred to Fitabase, a research platform that collects and manages data from internet-connected activity devices (Small Steps Labs LLC, San Diego, CA; available at https://www.fitabase.com). Fitabase does not collect personally identifiable information or IP addresses from synced participant devices. Fitbit accounts for each participant will be manually connected to the Fitabase platform and assigned a unique, non-identifiable study ID. All data associated with the Fitbit device will be linked to the allocated study ID in the Fitabase platform.

All paper records (i.e. consent forms, surveys, compliance and adherence logs, accelerometer register, clinical data) will be stored in locked filing cabinets located in the JHFMHN Research Unit. Consent forms and referral forms (see *Safety monitoring and reporting*) will be stored separately from hardcopies of the surveys. There will be restricted access to files, with access limited to project staff with direct involvement in the study. Paper records will be retained in line with *Ministry of Health Policy Directive PD2009_057 Records Management-Department of Health and the State Records Authority NSW General Retention and Disposal Authority–Public Health Services: Administrative Records-GDA21*.

Electronic files will be saved in limited-access folders, access to which requires a JHFMHN log-in, password and specific permissions. Data will be stored for five years from date of first publication of the data, in line with the recommendations of the National Health and Medical Research Council. After this time, all electronic files will be deleted and paper files shredded. All data will be deleted from Fitabase on completion or withdrawal from the project. Ninety days after the completion of data collection, Fitabase will remove the project from their data management system, disassociate the data from the project, and begin to remove access to the data by deleting it from its servers and backup systems.

### Statistical analysis

A detailed Statistical Analysis Plan will be developed and agreed upon by the project team prior to the commencement of data analysis.

#### Quantitative data

Quantitative feasibility outcomes will be reported descriptively and narratively. Rates of recruitment, retention, protocol adherence/compliance, intervention fidelity and adverse events will be presented as frequencies and proportions. Participants recruited to the study in the initial wave (January–March 2020), prior to the suspension of recruitment during the COVID-19 outbreak in NSW (March–June 2020), will be replaced by new participants to achieve the target sample size and will have their data excluded from the final analysis.

Primary and secondary outcomes will be analysed descriptively and presented for the overall sample and by group. Means, standard deviations and ranges, or medians and interquartile ranges, as appropriate, will be reported for continuous data, and frequency counts and proportions for categorical data, with associated 95% confidence intervals. Missing data for each of the primary and secondary outcome measures will be described using descriptive statistics. Missing data will not be replaced or imputed.

As this pilot study primarily aims to determine the feasibility of conducting a future definitive intervention trial and is not powered for statistical hypothesis testing, statistical analysis of the potential effectiveness of the intervention will not be performed on the clinical outcome data. Physical activity, cardiorespiratory fitness, and cardiometabolic risk data collected at each timepoint will be summarised descriptively for each group. Whilst between-group statistical comparisons will not be undertaken, post-intervention differences between groups will be reported using estimated mean differences and 95% confidence intervals, where appropriate.

Perceptions of exercise intensity, general health status and the study experience will be reported descriptively and narratively. All analyses will be performed using IBM SPSS software (Version 27) [40].

#### Qualitative data

Qualitative data on intervention acceptability obtained from the patient and staff evaluation surveys administered post-intervention will undergo thematic analysis, using both deductive and inductive coding methods [41], and iterative categorisation (IC). IC entails an iterative process of constant comparison between the data and emerging thematic categories [42]. This mixed-method approach to coding will entail using a priori codes derived from the evaluation surveys.

### Progression criteria

The decision to proceed to a definitive RCT will be informed by an assessment of feasibility outcomes according to a set of progression criteria determined a priori by the project team (Table 2). These criteria were developed utilising the traffic light system, as proposed by Avery et al. [43]. Green (go) indicates that the criteria have been met and that progression to the RCT is possible without changes to the design or implementation of the intervention, amber (amend) indicates that some changes should be made to the protocol, and red (stop) indicates that the RCT should not proceed.

**Table 2:** FIT HEART progression criteria

	Green	Amber	Red
Recruitment			
% of eligible patients consenting to participation	≥ 50%	30–49%	< 30%
Retention			
% of recruited participants retained at post-intervention data collection timepoint with valid primary outcome data	≥ 80%	70−79%	< 70%
Protocol adherence			
% of recruited participants who adhere to allocated intervention	≥ 70%	50–69%	< 50%
% of recruited participants who comply with wearing an accelerometer	≥ 90%	70–89%	< 70%
Fidelity of intervention delivery			
% of intervention components delivered as per protocol	≥ 90%	70–89%	< 70%

### Oversight and monitoring

Coordination and oversight of the trial will be provided by the JHFMHN Research Unit (Investigators SK, AL and JB). Data management will be overseen by AL and VA.

#### Safety monitoring and reporting

Safety monitoring and reporting procedures will be aligned with the requirements for clinical trials conducted in Australia [44] and in NSW Public Health Organisations in particular (*Office for Health and Medical Research Policy Directive PD2017_039-Safety Monitoring and Reporting for Clinical Trials Conducted in NSW Public Health Organisations*).

The occurrence of any unexpected or adverse events during the study period, irrespective of whether they may be causally related to the study, will be recorded. Daily monitoring of adverse events will be conducted via brief interviews with staff and patients and health record review. An adverse event that meets the criteria for a serious adverse event (SAE) will be reported to all relevant ethics committees: JHFMHN Human Research Ethics Committee (HREC), Aboriginal Health & Medical Research Council (AH&MRC). SAEs occurring after a subject has withdrawn from, or completed participation in, the study will not be reported unless the investigators believe that the event may have been caused by the study intervention or a protocol procedure. Investigators will determine potential causality based on whether there is a temporal relationship to participation in the study, as well as whether the event is unexpected or unexplained given the participant’s previous medical conditions and current medications.

Prior to each exergaming session, the Dynamic Appraisal of Situational Aggression (DASA) will be completed for each participant. The DASA is an instrument designed to assess the risk of imminent violence (i.e. within 24 h of administration) in psychiatric patient populations [45]. Any participant that presents a risk of aggression/violence will be excluded from the session for that day and will recommence sessions subject to re-appraisal of risk.

In cases where a participant discloses, or research staff are alerted to, a new and/or unmanaged health condition during the course of the study, a referral form will be completed and handed over to clinical staff at the study site for appropriate follow-up. Information alerting participants to this referral process is included in the PIS.

## Discussion

The patient and environmental factors of secure mental health units present unique challenges to the integration of conventional physical activity interventions into routine care. This protocol describes the design and methods of a non-randomised, two-arm pilot study assessing the feasibility, acceptability and potential effectiveness of implementing a novel exergaming-based intervention (FIT HEART) among patients of a secure mental health unit. Participants in the intervention group will receive a 12-week programme, comprised of three 30-min exergaming sessions per week, with those in the control group receiving routine care. The primary objective of the study is to determine whether the FIT HEART programme is a feasible intervention for improving physical activity levels, as measured by recruitment rates, retention in the programme, protocol adherence, and patient and staff acceptability ratings. Baseline to post-intervention changes in physical health outcomes (levels of physical activity; cardiovascular fitness; clinical measures of cardiometabolic risk) will also be assessed. The results will be disseminated in peer-reviewed journals and at national and international conferences and, if suggestive of intervention feasibility and acceptability, will inform a larger RCT of effectiveness, in which multiple follow-up points will be used.

### Strengths and limitations

A strength of the FIT HEART study is that it is the first to investigate the feasibility and acceptability of exergaming in a psychiatric inpatient setting and assess the potential impact of such an intervention on risk factors for cardiovascular and metabolic disease. Moreover, to our knowledge, it is the first study to trial an exergaming intervention in a correctional mental health facility.

This is a non-randomised and unblinded study and, as such, is potentially susceptible to bias and confounding, limiting the ability to draw causal inferences about the effectiveness of the intervention and generalisability of the findings to the target patient population. The study will be conducted in an inpatient setting, across two high-secure hospital wards, with patient placement according to level of acuity of psychiatric illness. Individual randomisation of participants to the control and intervention groups is not practical and will increase the risk of treatment contamination, as it will not be possible, within a particular ward, to ensure that participants in the control group avoid exposure to the intervention components. The use of a prospective design and a concurrent control group, however, will help to minimise selection bias associated with non-random allocation. Given the nature of the intervention, it is not feasible to blind participants and unit staff to the study conditions. By utilising objective measures of clinical outcomes (i.e. levels of physical activity, cardiovascular fitness, cardiometabolic risk), the risk of performance bias due to awareness of the intervention allocation will be reduced.

Impediments to recruitment and retention may impact on the sample representativeness and power to detect the primary outcomes of interest. In planning the recruitment strategy, bed capacity in each of the units, patient length of stay, and participant attrition has been considered. Nevertheless, attainment of the desired sample size is subject to patient flow through the unit and willingness to participate in the study. Recruitment may also be impacted by intermittent restriction of access to units and participants during periods when the unit is in lockdown due to staffing or security issues. A potential challenge for the study will be retaining participants for the duration of the 14-week assessment period due to unforeseen release or transfer from the Long Bay Mental Health Unit. In cases of release or transfer prior to week 12 of the study period, all efforts will be made to complete the “early discontinuation” assessments prior to discharge or in the week following transfer to another JHFMHN health centre.

The primary aim of the study is to determine the feasibility and acceptability of implementing the FIT HEART programme as an adjunct to routine care. Notwithstanding potential limitations and challenges, piloting the intervention in a real-world patient setting will contribute to an emerging evidence base and provide important information ahead of a more rigorous randomised controlled trial.

## Conclusion

The FIT HEART study will provide the first data, in Australia and internationally, on the feasibility, acceptability and potential effectiveness of exergaming as an alternative to routine care for mental health inpatients. Contributing to evidence-based practice in the care of patients with serious mental illness, this study will provide novel findings to inform cardiovascular health promotion and cardiovascular disease prevention strategies and addresses a clearly identified priority to reduce the excess health burden associated with cardiovascular disease among this patient population. More broadly, this research will facilitate delivery of optimal health services to a vulnerable population with complex physical and mental health needs and markedly elevated risk of cardiovascular disease.

## Study status

Recruitment and data collection commenced in January 2020, but was put on hold in March 2020 in response to the suspension of AH&MRC HREC approval for face-to for face-to-face data collection due to the emerging COVID-19 outbreak in NSW. Following a lifting of the suspension, recruitment recommenced in June 2020 and is anticipated to end in December 2022. Due to the nature of the intervention, requiring face-to-face data collection, there were no variations to the protocol with respect to the study design and methods. The study is being conducted in accordance with version 3.0 of the FIT HEART study protocol (14 November 2018).

## Trial sponsor

This study is sponsored by the Justice Health and Forensic Mental Health Network (JHFMHN), 1300 Anzac Parade, Malabar, New South Wales 2036. The study investigators are employed at the JHFMHN Research Unit. Beyond the study investigators, JHFMHN has had no role in the design or conduct of the research.

## Supplementary Information

**Additional file 1.** SPIRIT 2013 Checklist: Recommended items to address in a clinical trial protocol and related documents.

## Data Availability

Not applicable.

## Abbreviations

AH&MRC: Aboriginal Health and Medical Research Council
APSS: Adult Pre-Exercise Screening System
BMI: Body mass index
CAPE: Community Assessment of Psychic Experience
DASS: Depression Anxiety Stress Scale
DASA: Dynamic Appraisal of Situational Aggression
FIT HEART: Feasibility of an Intervention Targeting Health through Exergaming as an Alternative to Routine Treatment
HDL: High-density lipoprotein
HREC: Human Research Ethics Committee
IBM SPSS: IBM Statistical Package for the Social Sciences
IC: Iterative categorisation
ID: Identifier
JHFMHN: Justice Health and Forensic Mental Health Network
LDL: Low-density lipoprotein
MetS: Metabolic syndrome
MET: Metabolic equivalent of task
NSW: New South Wales
PIS: Participant Information Sheet
RCT: Randomised controlled trial
RPE: Rate of perceived exertion
SAE: Serious adverse event
SIMPAQ: Simple Physical Activity Questionnaire
SPIRIT: Standard Protocol Items: Recommendations for Interventional Trials
UBACC: University of California, San Diego Brief Assessment of Capacity to Consent
